# Correction to Brigatinib in Japanese patients with *ALK* ‐positive non‐small‐cell lung cancer: Final results of the phase 2 J‐ALTA trial

**DOI:** 10.1111/cas.16000

**Published:** 2023-12-10

Tatsuya Yoshida, Toru Kumagai, Ryo Toyozawa, Ryohei Katayama, Makoto Nishio, Takashi Seto, Koichi Goto, Nobuyuki Yamamoto, Yuichiro Ohe, Kentarou Kudou, Takayuki Asato, Pingkuan Zhang, Kazuhiko Nakagawa. Brigatinib in Japanese patients with ALK‐positive non‐small‐cell lung cancer: Final results of the phase 2 J‐ALTA trial. *Cancer Sci*. 2023;114:3698–3707. doi:10.1111/cas.15888.

The corrected Figure 2D and related statement in the legend is shown here:

**FIGURE 2:**
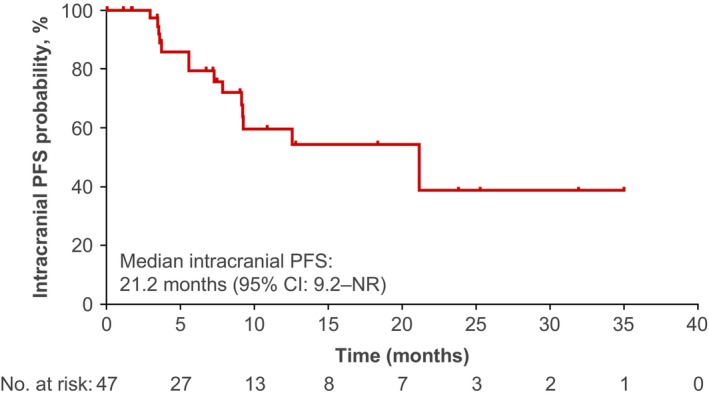
**Efficacy in the main cohort (post‐alectinib ± crizotinib; n = 47).** (A) DoR in patients with IRC‐assessed confirmed response. (B) PFS per IRC‐assessment. Twenty‐nine of 47 patients had events. (C) OS. At study end, 22 (47%) of 47 patients had died. (D) Intracranial PFS in all patients, regardless of intracranial disease status at baseline. In total, 15 of 47 patients had events of intracranial progression or death. For Kaplan–Meier estimation of intracranial PFS, systemic PD followed by intracranial PD was considered an event, whereas patients who had systemic PD and withdrew from the study without intracranial PD were censored. Abbreviations: DoR, duration of response; IRC, independent review committee; OS, overall survival; PD, progressive disease; PFS, progression‐free survival.

**FIGURE 3B:**
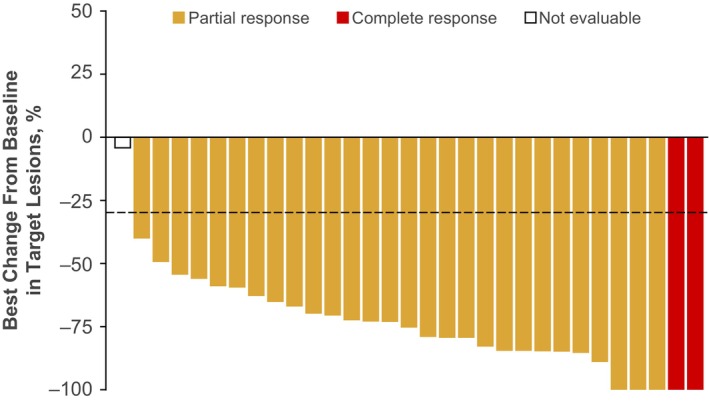


The corrected Figure 3B is shown here:

Errors in Figure S3 have been corrected in the Appendix S1 in the online Supporting Information section.

We apologize for this error.

